# Quantity and quality of care and staff knowledge regarding people with Parkinson’s disease in long-term nursing care: “real-life” results from the German Care4PD study

**DOI:** 10.3389/fnagi.2025.1701254

**Published:** 2026-01-02

**Authors:** Odette Fründt, Verena Caroline Lamb, Anne-Marie Hanff, Tobias Mai, Christiane Kirchner, Ali Amouzandeh, Carsten Buhmann, Rejko Krüger, Alfons Schnitzler, Martin Südmeyer

**Affiliations:** 1 Department of Neurology, Klinikum Ernst von Bergmann, Potsdam, Germany; 2 Transversal Translational Medicine, Luxembourg Institute of Health (LIH), Strassen, Luxembourg; 3 Department of Nursing Development/Nursing Research, University Hospital Frankfurt, Frankfurt, Germany; 4 Department of Neurology, University Medical Center Hamburg-Eppendorf, Hamburg, Germany; 5 Luxembourg Centre for Systems Biomedicine (LCSB), University of Luxembourg, Belvaux, Luxembourg; 6 Parkinson Research Clinic, Centre Hospitalier de Luxembourg (CHL), Luxembourg, Luxembourg; 7 Department of Neurology, University Medical Center Düsseldorf, Düsseldorf, Germany

**Keywords:** Parkinson’s disease, quantity, quality, staff knowledge, long-term care, nursing

## Abstract

**Introduction:**

Approximately 20% of people with Parkinson’s disease (PwP) in Germany need professional long-term care (LTC). Previous data have indicated a rather poor LTC situation and the need for more profound analyses. Therefore, we aimed to assess the quantity and quality of LTC care for PwP and the knowledge on Parkinson’s disease (PD) in German LTC nursing staff.

**Methods:**

Data from our nationwide, cross-sectoral Care4PD survey, which was distributed postally and online, were analyzed. Out of 295 completed anonymous LTC nurse questionnaires, 288 were included, with descriptive results presented in this study.

**Results:**

In terms of age and work experience, a representative sample of 288 participants, the majority (79%) of whom were registered LTC nurses, participated in the study. A total of 95% of them had certain experience with people with Parkinson’s disease (PwP). On average, each nurse supported approximately three PwP per week, with a mean care time of 48 min per day. A total of 17% of participants complained about “never” having enough staff, and 50% complained about “frequently changing” LTC personnel in their institution. Additionally, 10% reported “unsafe” care quality, with the occurrence of avoidable complications. Insufficient knowledge on PD and the importance of PD-specialized training were highlighted, with current training options often not recognized. Optimization suggestions consisted of more personnel and time capacities, educational measures, and interprofessional exchange.

**Discussion/conclusion:**

Improving PwP care in German LTC facilities requires not only the general provision of more personnel and time resources but also, in particular, the development of greater expertise among LTC nursing staff to optimize care quality. The existing, but little-known, training opportunities should therefore be made known to a larger number of LTC nurses.

## 1 Introduction

In Germany, out of over 400,000 people suffering from idiopathic Parkinson’s disease (PD; Deutsche Gesellschaft für Parkinson und Bewegungsstörungen, n.d.; Heinzel et al., 2018), approximately half of them show advanced disease stages, according to the Hoehn and Yahr scale (H&Y), with a score of ≥3 (von Campenhausen et al., 2005). At this stage, several motor and non-motor symptoms significantly impact patients’ quality of life (Kurihara et al., 2020; Sanchez-Luengos et al., 2022). Following dementia, out of all patients with chronic diseases aged >65 years, people with Parkinson’s disease (PwP) have the highest risk of becoming care-dependent (van den Bussche et al., 2014). In Germany, nearly 40% of PwP have a care degree, and approximately one in every five PwP needs professional long-term care (LTC) (Fründt et al., 2022). The latter is primarily provided by mobile (outpatient) nursing services, followed by residency in nursing homes and professional domestic 24-h care (Fründt et al., 2021). A recent scoping review highlighted the rather poor care situation of PwP in such LTC facilities (van Rumund et al., 2014; Weerkamp et al., 2014; Safarpour et al., 2015; Fründt et al., 2022) and the need for more detailed analyses of the care situation in these institutions due to the lack of data (Fründt et al., 2022). LTC nursing staff’s knowledge of PD in Germany has previously been found to be rather poor, emphasizing the need for more education, at least in nursing homes (Mai and Ketter, 2021). Furthermore, no data are available on mobile nursing services, which represent the most frequently used type of LTC (Fründt et al., 2021). In Germany, there are several educational training options available for nursing staff to improve their knowledge regarding PD. These options have been summarized earlier and range from a basic level (“Online care school Parkinson” or “Parkinson care specialist”) to higher specialized qualifications, such as Parkinson’s assistants (“PASS”) for outpatients and Parkinson’s nurses (“PD nurses”) for inpatient settings (Prell et al., 2020; Fründt et al., 2023b). To date, no data on how often these qualifications are used in LTC settings are available.

Consequently, this study used a nationwide anonymous survey, distributed to participants from all 16 federal states of Germany, to evaluate the perspectives of LTC nursing staff, with the aim of examining the quantity and quality of professional LTC in different settings, as well as their knowledge about PD.

## 2 Materials and methods

We analyzed data from our nationwide, cross-sectoral, survey-based Care4PD study (Thiemann Stiftung, 2021), which has been previously described by Fründt et al. (2021). The survey consisted of two questionnaires developed to evaluate the care situation of PwP, with a focus on LTC. In this study, we report the results from nursing staff of LTC facilities regarding their evaluation of the quantity and quality of LTC nursing care, as well as their knowledge about PD and their need for PD-specific education or training. Study participation was voluntary and anonymous. Single- or multiple-choice questions, graduated (Likert) scales, visual analog scales (from 0 = not applicable/not at all to 10 = very applicable/very much), and open questions were used (see Supplementary material). Data collection was performed between August and December 2021.

### 2.1 LTC nursing staff questionnaire

This questionnaire, which consisted of 32 questions in total, was addressed to the nursing staff of LTC facilities. After a testing phase (which included interviews with five nurses) and a consultation with a statistician, the questionnaire was revised, condensed, and optimized to the final version. The questionnaires were distributed nationwide using a link to the online version [created with the online survey tool Unipark by Tivian (15)], which was sent to nearly 900 institutions (nursing associations [“Pflegeverbände”], nursing agencies [“Pflegeträger”], labor unions [“ver.di”], and supervisors of LTC facilities), who then forwarded it to their members. We also provided a written version that was distributed via post directly to another 960 randomly selected LTC facilities (7,000 copies; mobile nursing services, nursing homes, and 24-h care) and the nursing staff working there.

Returned questionnaires were processed identically to our patient survey that was introduced earlier (Fründt et al., 2021, 2023a,b), which means that Unipark results were first exported into Excel to later create an SPSS-compatible database. Questionnaires with inconsistent or >30% missing data (*n* = 7) were excluded from analysis.

### 2.2 Statistical analysis

The questionnaire results are presented as the number (*n*) and percentage of (%) participants, or means with standard deviation (SD), and range from minimum to maximum. Inner-group comparisons were performed between two subgroups of services, “nursing home” and “mobile nursing,” using Student’s *t*-test for metrical variables (or corrected *p*-values according to the Welch Test in case of unequal variances), the Mann–Whitney *U*-test for categorical variables, or Pearson’s chi-squared test for binary variables. Correlations were calculated using Spearman’s rho.

## 3 Results

### 3.1 Group characteristics and nurses’ experiences with PwP

A total of 295 questionnaires were returned, of which 105 were completed online. The response rate was 11.7% for the online version (105/900) and 2.7% for the paper version (190/7000), with a total response rate of 3.7% (295/7900). Of the 295 returned nursing staff questionnaires, 288 were finally included. The characteristics of the participants are described in Table 1.

**Table 1:** Nursing staff group characteristics.

Parameter	Nursing staff (all; *n* = 288) Mean [Min–Max] ± SD	Number of participants
Age (years)	41.9 [18–66] ± SD 11.7	*n* = 286
Gender	♂ 20.8% ♀ 78.8% divers: 0.3%	*n* = 60 *n* = 227 *n* = 1
Type of LTC facility	Mobile nursing service: 17.7% Nursing home: 60.8% Others*: 9.6% Multiple answers: 11.5% (n.a.: 0.3%)	*n* = 51 *n* = 175 *n* = 28 *n* = 33 *n* = 1
Nursing staff’s mother tongue	German: 91.7% Others: 8.0% (Polish) (n.a.: 0.3%)	*n* = 264 *n* = 23 *n* = 1
Qualification level	Exam nurse school: 79.5% University (BA/MA): 7.3% Care assistant/training: 11.5% (n.a.: 1.7%)	*n* = 229 *n* = 21 *n* = 33 *n* = 5
Working experience (years)	17.7 [1–45] ± SD 11.3	*n* = 288
Working condition	Full-time: 72.6% Part-time: 25.7% Temporary: 0.7%	*n* = 209 *n* = 74 *n* = 2
Care of PwP (experience)	Currently: 63.2% Previously: 32.3% Never: 3.1% Do not know: 1.0% (n.a.: 0.3%)	*n* = 182 *n* = 93 *n* = 9 *n* = 3 *n* = 1
Priority care for PwP in LTC institution	Yes: 4.2% No, but desirable: 44.4% No, no need for: 38.2% Do not know: 12.2% (n.a.: 1%)	*n* = 12 *n* = 128 *n* = 110 *n* = 35 *n* = 3

Results of the group characteristics are given as mean [minimum–maximum] and standard deviation (SD).

PD, Parkinson’s disease; LTC, long-term care; PwP, people with PD; *n*, number; n.a., no or multiple answers; SD, standard deviation.

*Other affiliations: day or night care, assisted living, 24-h care, flat-sharing care. Care assistant = “Pflegeassistent”.

The nursing staff group was predominantly female and middle-aged, with a mean age of approximately 42 years, ranging from the legal age of majority to retirement age. The majority of participants (79%) held a registered qualification level (completing an examination after a 3-year course of nursing school) and had sufficient working experience, with an average age of 18 years. A total of 8% of the participants spoke a mother language other than German, with Polish being the most common. Nearly a quarter (26%) of the participants worked part-time or under temporary work contracts (travel nurse = “Zeitarbeit”). Only a small number of institutions focused on the care of PwP as a priority, but nearly 44% of participants expressed a desire for such a focus.

**Figure 1:**
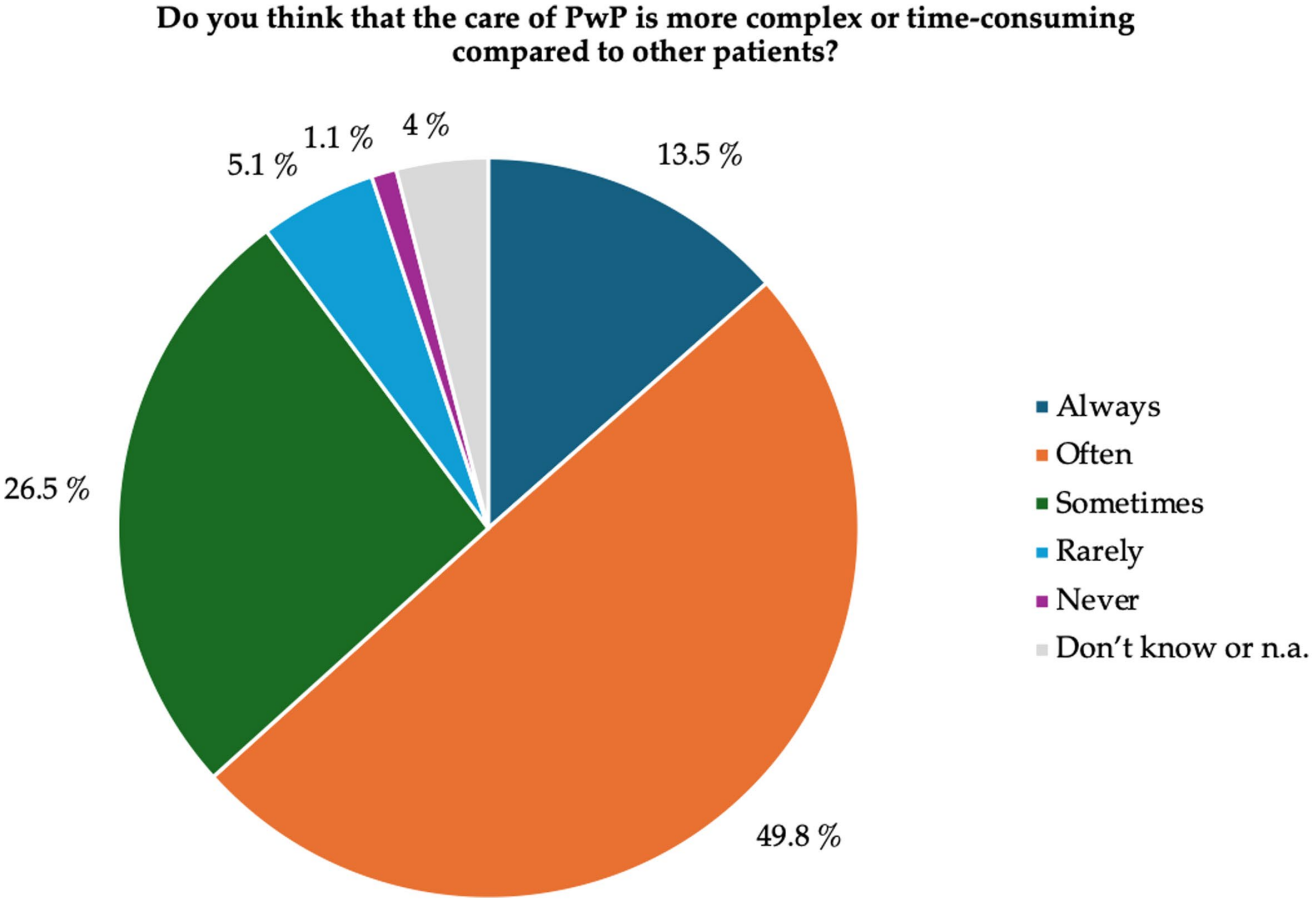
Complexity and time consumption of nursing care for PwP. Results are given as percentages of participants. PwP, people with Parkinson’s disease; n.a., no or multiple answers.

A total of 95.5% (*n* = 275) of the nursing staff members reported having had current or previous contact with PwP. These individuals were included in the subsequent analyses. When asked about the complexity and time consumption of care for PwP, more than 60% of the participants assessed this aspect as always or often high (see Figure 1).

**Figure 2:**
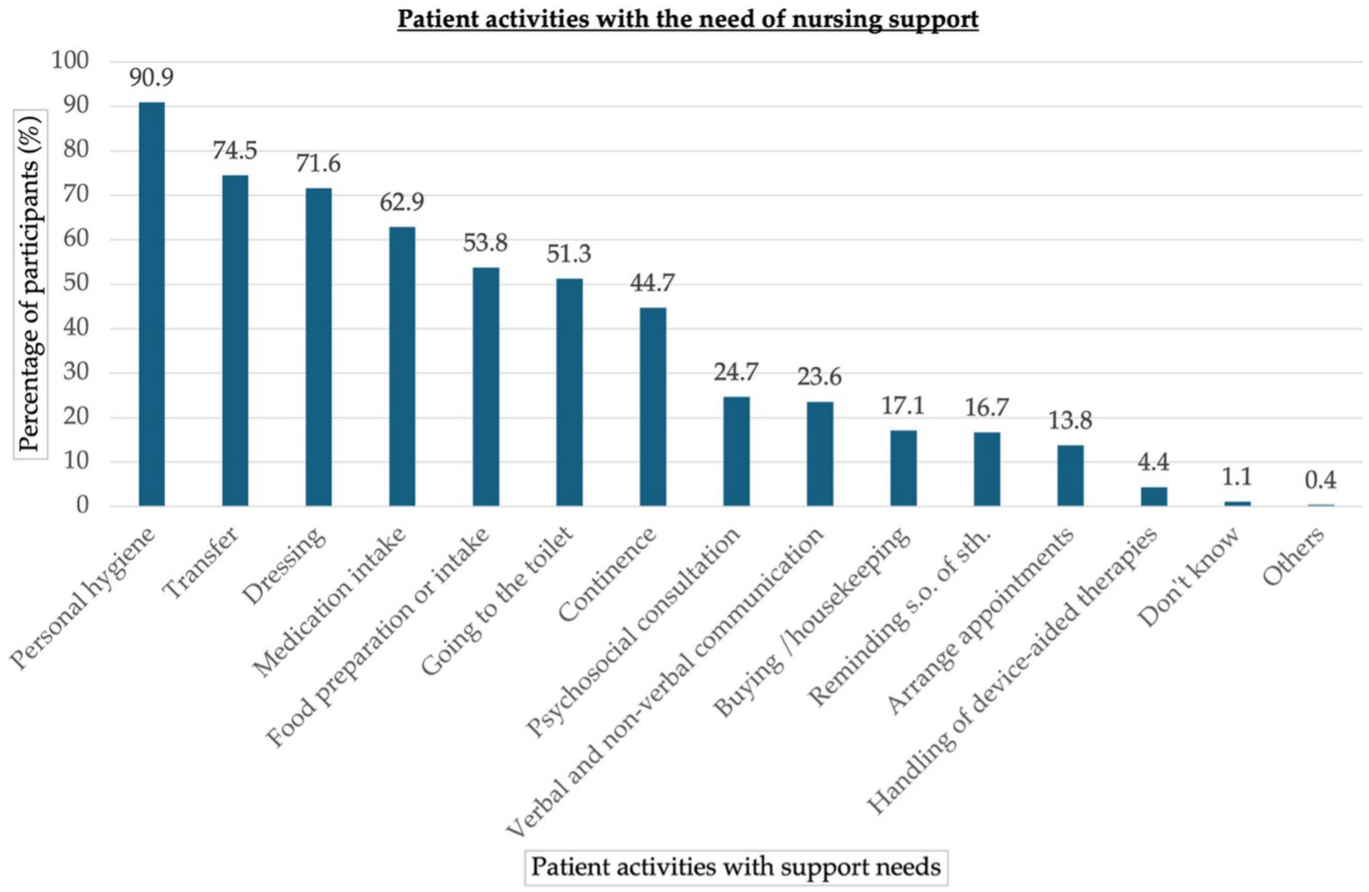
Support of PwP activities. Results are given as percentage of participants. PwP, people with Parkinson’s disease; n.a., no or multiple answers.

According to the nursing participants, PwP need most support with personal hygiene, transfer, dressing, and medication intake, whereas the handling of device-aided therapies was considered less important (see Figure 2).

### 3.2 Quantity and quality of nursing care in LTC facilities

For the following analyses, only those participants with current or previous experience with PwP were included (*n* = 275).

The indicators used to assess nursing care quantity and quality in LTC facilities are given in Table 2.

**Table 2:** Indicators for quantity and quality of nursing care at long-term care facilities.

Parameter	Nurses experienced with PD (*n* = 275^*^) Mean [Min–Max] ± SD	Number of participants
Number of PwP to care for per week	3 [1–15] ± SD 2.3	*n* = 91
Estimated care time in minutes	All: 48 [5–300] ± SD 41.0 MNS/NH: 45 [5–300] ± SD 42.3	*n* = 74 *n* = 58
Timely intake of medication (0 = never to 10 = very timely)	7.5 [0–10] ± SD 2.4	*n* = 260
Sufficient nursing staff	Always: 4% Often: 24% Sometimes: 28.7% Occasionally: 25.5% Never: 17.5% (n.a.: 0.4%)	*n* = 11 *n* = 66 *n* = 79 *n* = 70 *n* = 48 (*n* = 1)
Frequently changing personnel	Always: 4% Often: 44.4% Sometimes: 32.7% Occasionally: 17.8% Never: 1.1%	*n* = 11 *n* = 122 *n* = 90 *n* = 49 *n* = 3
Permanent contact person	Always: 26.2% Often: 40.7% Sometimes:20.0% Occasionally: 12.7% Never: 0.4%	*n* = 72 *n* = 112 *n* = 55 *n* = 35 *n* = 1
Sufficient support	Always: 6.5% Often: 49.8% Sometimes: 28.7% Occasionally: 10.5% Never: 0.7% (do not know/n.a.: 3.6%)	*n* = 18 *n* = 137 *n* = 79 *n* = 29 *n* = 2 (*n* = 10)
Quality of care^#^	Optimal care: 8.4% Adequate care: 50.2% Safe (routine) care: 27.3% Unsafe care: 9.8% (do not know/n.a.: 4.4%)	*n* = 23 *n* = 138 *n* = 75 *n* = 27 (*n* = 12)
Overall care situation of PwP in LTC facility (0 = insufficient to 10 = optimal)	5.9 [0–10] ± SD 2.2	*n* = 225

Results are given as mean [minimum–maximum] and standard deviation (SD). *Only nursing staff with current or previous experience with PwP were included. ^#^According to Fiechter and Meier (1998).

PD, Parkinson’s disease; PwP, people with PD; LTC, long-term care; MNS, mobile nursing service; NH; nursing home; *n*, number.

Regarding care quantity, the nursing staff group reported that they support, on average, three PwP per week, with a maximum of 15 PwP per nurse. Nursing care time was estimated to be approximately 48 min per day when including all types of care (especially 24-h care). When focusing on the care settings of mobile nursing services (MNS) and nursing homes (NH), care time was similar between the two settings (*p* > 0.05). The timely intake of medication was generally ensured, with a score of 7.5 on a scale from 0 = never to 10 = very timely. Having a sufficient number of nursing staff members was only reported by approximately one-quarter of the nursing participants (“always” or “often” combined), while 17% complained about “never” having enough LTC personnel. Nearly half of the nursing staff reported “frequently changing” nursing staff. However, approximately two-thirds of the participants reported having a permanent nursing contact person for PwP, and half of them felt that the nursing support available was sufficient (“always” or “often”).

Quality of care was ranked as “adequate” by most of the nursing participants; however, nearly 10% reported “unsafe” care quality at their institution, with the occurrence of avoidable complications. The overall care situation was rated as moderate, with a ranking of 6 on a scale from 0 = insufficient to 10 = optimal.

### 3.3 Knowledge of LTC nursing staff on PD

The nursing staff’s self-ratings on their knowledge of PD are shown in Table 3 and reveal rather insufficient knowledge on PD symptoms and therapy, especially regarding the handling of advanced device-aided therapies (DBS, pumps). This pattern was even more pronounced when including all participants (*n* = 288), even those without recent or past contact with PwP (PD symptoms: mean of 6.5 [0–10] ± SD 2.1; PD therapy in general: mean of 4.0 [0–10] ± SD 2.0; handling of device-aided therapies: mean of 2.4 [0–10] ± SD 2.4).

**Table 3:** Nursing staff’s specific knowledge on Parkinson’s disease at LTC facilities.

Parameter	Nurses experienced with PD (*n* = 275^*^) Mean [Min–Max] ± SD	Number of participants
PD symptoms	6.7 [0–10] ± SD 2,0	*n* = 274
PD medication	6.1 [0–10] ± SD 2,2	*n* = 274
Handling of DBS	2.4 [0–10] ± SD 2,4	*n* = 273
Handling of medication pumps	2.4 [0–10] ± SD 2,8	*n* = 273

Results are given as mean [minimum–maximum] and standard deviation (SD). Participants rated their knowledge on a scale from 0 = “none” to 10 = “profound”.

PD, Parkinson’s disease; DBS, deep brain stimulation; *n*, number.

**Figure 3:**
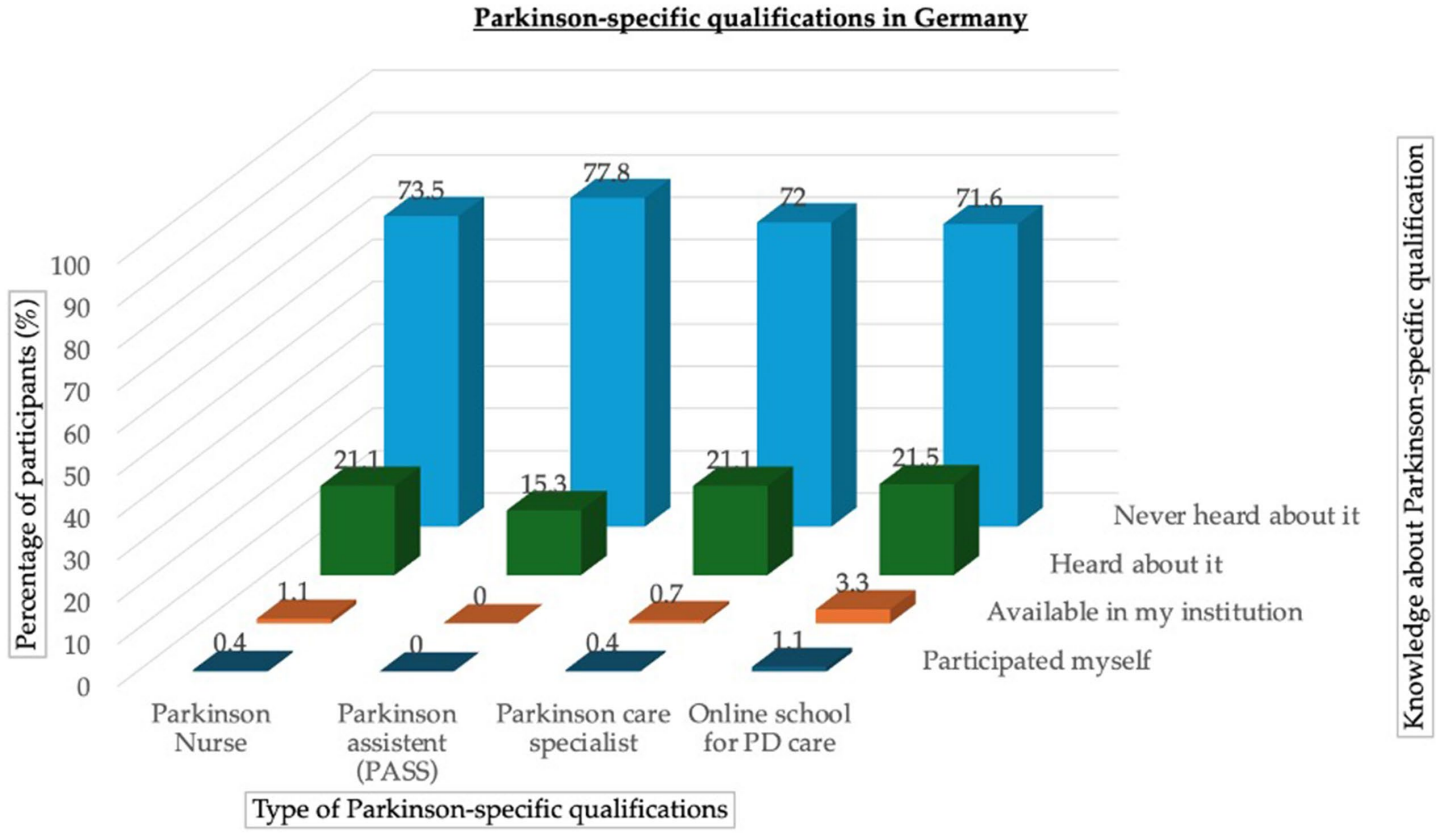
Deployment of Parkinson-specific qualifications in long-term care in Germany. The four existing qualification options, ranking from specialized (PD nurse, PASS) to more basic qualification levels (Parkinson’s care specialist, online school for Parkinson’s care), are shown with the percentages of participants (%) knowing of and/or using them.

The majority of participants (>70%) did not know about the existence of PD-specific nursing qualifications (PD nurse, PD assistant, PD care specialist, and online school for PD care). Nearly 3% of the participants’ facilities employed nursing staff with basic PD-specific care qualifications (achieved through an online school for PD care). Specialized PD nurses were only available at 1.1% of facilities (see Figure 3).

Nursing staff members rated the importance of proper PD-specialized training as rather important (mean of 7.0 ± SD 2.9 [0–10] on a scale from 0 = not at all important to 10 = very important). When looking at differences among the LTC settings, the need for nurses specialized in PD care was rated higher in the NH (mean of 5.7 ± SD 3.3 [0–10], *n* = 46) than in the MNS (mean 7.7 ± SD 2.6 [0–10], *n* = 159) subgroup. Further subgroup analyses (MNS vs. NH) revealed no other relevant differences among the groups.

### 3.4 Suggestions for how to optimize LTC in PD

**Figure 4:**
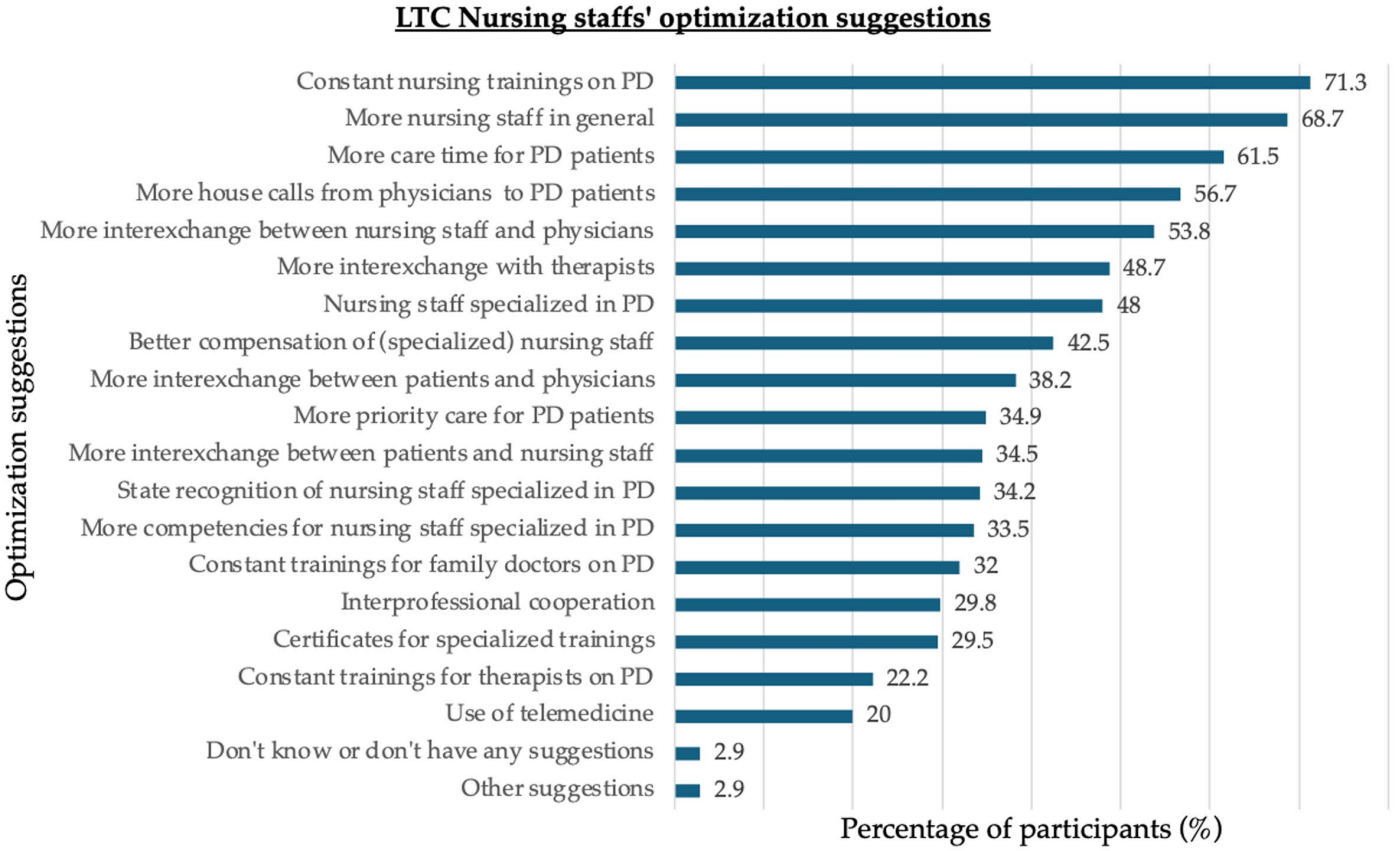
Optimization suggestions by long-term care nursing staff. Optimization suggestions of participants given as % of participants, in a ranked order.

Figure 4 provides an overview of the participants’ suggestions (based on predefined, study-developed response options, as well as free text options) for how to optimize LTC for PD. Personnel and time resources, as well as educational aspects and exchange between nurses and physicians, were predominantly requested.

### 3.5 Correlations between care quantity/nursing staff knowledge and care quality

There was a significant negative correlation (*r* = −0.416, *p* < 0.001) between the staffing numbers (ranging from 1 = “never” to 5 = “always” enough) and care quality evaluations (ranging from 1 = “optimal” to 4 = “unsafe”), revealing that care quality improves with adequate staffing levels. In this study, there was no significant correlation between the staff’s time capacity per person in minutes per day and care quality (*r* = −0.25, *p* = 0.839). The same was true for the LTC nursing staff’s (overall) knowledge on PD, ranging from 0 = none to 10 = profound, and its correlation with care quality (r = −0.99, *p* = 0.109).

## 4 Discussion

Our Care4PD study is the first to examine indicators for the quantity and quality of nursing care of PwP in Germany and presents data on the knowledge of LTC nursing staff on PD and their need for and interest in training to specialize in PD. The overall low response rate of 3.7% lies within the reported range of previous studies (L'Ecuyer et al., 2023), which will be discussed below. Acceptance to response was higher online (11.7%) compared to the written version (2.7%).

### 4.1 Group characteristics

As shown in Table 1, we included a representative group of nurses, who were mostly well-qualified, with a wide range of ages and work experience. This allowed us to obtain an impression of the overall “everyday life” situation. The nursing participants in this study primarily worked in nursing homes (NHs), but the subgroup analyses revealed no relevant differences between settings besides the greater need for PD-specialized nurses in nursing homes (NHs). Therefore, we believe that the general care situation is similar for most PwP receiving professional care in “classical settings,” such as NHs or mobile nursing services (MNS), where the majority of PwP are cared for Fründt et al. (2021). An exception may be other care settings, such as 24-h care, which may benefit from longer and more intensive care, but this aspect cannot be examined further here due to the small sample size of this subgroup.

### 4.2 Quantity and quality of nursing care in LTC facilities

A majority of participants (95.5%) had contact/experience with PD patients in the past or present and took care of approximately three PwP per week. Therefore, PD seems to be a typical patient profile in LTC facilities, again underlining the relevance of PD in this setting (Mitchell et al., 1996; Hoegh et al., 2013; Fründt et al., 2022). The need for care was especially high for basic activities of daily living, such as personal hygiene, transfer, and dressing, which are all rather complex and time-consuming activities, especially when factoring in the psychomotor slowness of most PwP (Vlagsma et al., 2016). This might explain why nurses in our study rated the care of PwP as rather complex and time-consuming compared to other LTC patient groups.

Regarding the quantity of care, our results indicate a relatively low mean care time of nearly 50 min per PwP per day, regardless of the setting (mobile nursing service vs. nursing home). This timing is very poor compared with data from a previous study, which showed that families/relatives who cared for PwP, before they transitioned into institutionalized care, provided care for approximately 7.6 h per day (Jensen et al., 2021). The reason for the time gap remains unclear: do the families/relatives still provide the biggest support, or do PwP have unfulfilled needs that are not being addressed? The latter assumption might explain the previous finding that, after the institutionalization of PwP in nursing homes, caregiver burden (and care time) of their relatives decreased, but this was accompanied by the disadvantageous outcome of clinical worsening of the institutionalized PwP (Jensen et al., 2021). Therefore, future nursing care settings should provide more time and personnel resources to overcome this care gap.

Speaking of personnel resources, the current study provides concrete data on predominantly insufficient rates of nursing staff, with 17% of participants complaining about “never” having enough personnel at their institution. This may be a result of the nationwide nursing shortage in Germany, where a lack of nearly 280,000 to 690,000 nurses is expected until 2049 (Destatis, 2024). However, it can be inferred that, although both groups reported about frequently changing nursing staff, PwP are often still provided with a permanent nursing contact partner. This is important for a good nurse–patient partnership, which “takes time” to develop. As highlighted by Yuan and Murphy, such a partnership can ultimately improve the “quality of service, treatment outcomes, clients’ safety and satisfaction” and “can [furthermore] encourage clients’ self-management and improve person-centered nursing care” (Yuan, 2019).

Quality of care was primarily ranked as “adequate” by our participants. However, nearly 10% of nursing staff reported the care quality at their institution as “unsafe,” defined as the “occurrence of avoidable complications,” which indicates the need to address this issue. One solution, according to requests from our participants, could be the provision of proper nursing staff numbers and sufficient time resources. Although time resources did not significantly correlate with care quantity in this study, adequate staffing levels were significantly correlated with care quality. This could prevent care complications, as previous studies have found that nursing homes with high numbers of registered nurses had lower rehospitalization and emergency department visit rates (Tamburello, 2023). This, in turn, could increase the quality of life of PwP while also reducing economic burdens, as care complications may result in higher morbidity and hospitalization rates (Audet et al., 2018; Choi et al., 2021), which could actually be avoided.

Finally, the overall care situation was rated as moderate (6 on a scale from 0 = insufficient to 10 = optimal), with some potential for improvement.

### 4.3 Knowledge of LTC nursing staff on PD

Our results indicate rather insufficient knowledge of LTC nursing staff on PD symptoms and therapy, especially regarding the handling of advanced device-aided therapies. This might be explained by the fact that neurologic topics—particularly PD—are mentioned only marginally in the current general nursing education program in Germany (Fründt et al., 2023a,b). To address this, integrating certain “minimal knowledge” on PD into the basic education of nursing staff or offering specific Parkinson’s education programs (Chenoweth et al., 2013) could be helpful in the future. Furthermore, structured and comprehensive training on LTC roles could further equip nurses with the special knowledge needed regarding relevant neurological diseases in LTC settings.

Additionally, the majority of participants did not know about the existence of trained nursing staff specialized in PD, and the majority of LTC facilities did not use them. This is noteworthy, as advanced training options, such as PD nurse training, have been implemented in Germany since 2006 (Mai, 2018). Other options, such as the online school for Parkinson’s care, are easily accessible online, free of charge, and have low admission requirements. These results are comparable to a previous study showing that LTC facilities rarely know about the existence of specialized PD nurses, and they were not a part of LTC teams for a long time (Mai and Ketter, 2021). However, similar to the findings of Mai and Ketter (2021), our study revealed that the need for specialized LTC nursing staff was rated relatively high. This need was considered even more important in the nursing home environment compared to mobile nursing services, according to the ratings. To overcome this challenge, the upcoming curriculum for PD nurse training, which has been restricted to inpatient settings for a long time, plans to also approve LTC staff participation starting in 2026. Additionally, the existing training options (PD nurse, PASS, PD specialist, and online school for Parkinson’s care) should be advertised more extensively to reach more LTC personnel. We found that 7% of participants in this study had a university degree. Developing academic continuing education programs for this target group should be considered. Furthermore, providing training and educational material on PD in other languages (e.g., Polish, Russian) would be helpful, as we found a relatively high number of nursing participants (8%) from foreign countries. According to the World Health Organization (WHO), findings of the State of the World’s Nursing 2025 (SoWN) report suggest that “1 in 7 nurses worldwide […] are foreign-born, highlighting reliance on international migration” (WHO, 2023; World Health Organization-Regional Office for Europe, 2025).

Overall, regarding the optimization suggestions of our participants, time and personnel resources, training options, and interprofessional exchange were rated as more important than, for example, state accreditation, certification of training courses, or broader competency frameworks. Therefore, prioritizing the basic working environmental settings is needed to optimize the nursing care situation of PwP, and likely for all people receiving LTC.

### 4.4 Limitations

Compared with previous studies (L'Ecuyer et al., 2023), response rates were very low. This might raise concerns about a non-responder bias, a response bias (e.g., those nurses who are generally satisfied with their work conditions did not return the survey, whereas negative responses predominated), or the generalizability of study results. It is a well-known phenomenon that—although nurses make up the largest healthcare profession—response rates to nursing surveys are declining. Possible reasons as well as strategies to increase participation rates were discussed earlier (VanGeest and Johnson, 2011; O'Connor, 2022; L'Ecuyer et al., 2023; Timmins et al., 2023). Another distribution method, e.g., via directly contacting nurses (Corner and Lemonde, 2019) or limiting the survey volume, could possibly increase response rates of future studies.

Furthermore, only nursing staff members were addressed in this study to evaluate LTC quality and quantity. However, further analyses of patient responses based on our comprehensive Care4PD patient survey (Fründt et al., 2021; Fründt et al., 2023a,b) are expected soon (work in progress). Assessments of caregivers would also be an interesting prospect.

Another limitation that could influence study results might be the poor experience and knowledge of nursing staff regarding PD. The latter finding is in line with the findings of previous studies in hospital nurse settings (Chenoweth et al., 2013) and also with other primary healthcare providers, such as general practitioners, who similarly show notable PD knowledge gaps (Thompson et al., 2013), suggesting that multidisciplinary education programs should also be considered (Lennaerts-Kats et al., 2022; Fornes-Romero et al., 2024).

## 5 Conclusion

Data from nursing staff members indicated that PwP are supported by LTC nursing staff on a regular basis. However, personnel and time resources are low, with sparse and frequently changing nursing staff, although relatively stable permanent contact partners exist. Care quality was rated as only moderate, with 10% of care reported as unsafe and leading to avoidable complications. This highlights a care situation that requires improvement. The expertise of nursing staff with respect to PD is rather low, especially regarding PD therapies and the existing advanced training options (e.g., PD nurses); however, the interest in such training options was rated as high.

In addition to analyzing the special needs of PwP in LTC and providing more personnel and time resources in general, encouraging more expertise in LTC nursing staff through training could help to optimize the care situation of PwP who require LTC.

## Data Availability

The raw data supporting the conclusions of this article will be made available by the authors, without undue reservation.
