# Supplementary material for: Quantity and quality of care and staff knowledge regarding people with Parkinson’s disease in long-term nursing care: “real-life” results from the German Care4PD study
Source: Front Aging Neurosci. 2026 Jan 2;17:1701254. doi: 10.3389/fnagi.2025.1701254 (PMC12808439; doi:10.3389/fnagi.2025.1701254)
Supplement: Supplementary file 1 [file Data_Sheet_1.PDF]

Supplementary material S1

<u>Selection of questions of the comprehensive Care4PD questionnaire used for this analysis</u>

1. **Your gender?** ▢ Male ▢ Female ▢ Diverse

2. **How old are you?** ___________ years

3. **Your mother language?** ▢ German ▢ Others: ___________ (Please term)

4. **Since when have you been working as a caregiver in elderly or healthcare?** ___________ years

5. **In which area of elderly or nursing care are you currently working?**

▢ Mobile nursing service ▢ Day- or nighttime care facility

▢ Assisted living with nursing care ▢ Nursing home

▢ 24-hour care ▢ Care shared living community

▢ Others: ___________ (Please term)

6. **Which degree(s) do you have?**

▢ Exam: Elderly Care Training (until 2019) ▢ Exam: Health/Nursing Care Education

▢ Exam: Generalist nursing training (since 2020) ▢ Advanced Practice Nurse

▢ Bachelor in Nursing care ▢ Bachelor/Master or specialization gerontology

▢ Bachelor/Master or specialization palliative care ▢ Care assistant or still in training

▢ Others: ___________ (Please term)

7. **What is your current employment status?**

▢ Full-time ▢ Part-time ▢ Temporary work concepts

▢ I do not want to provide any information about that

8. **From your point of view: Does your facility have an adequate number of nursing staff?**

▢ Always ▢ Often ▢ Sometimes ▢ Occasionally ▢ Never

9. Are your patients cared for by frequently changing nursing staff?

☐ Always ☐ Often ☐ Sometimes ☐ Occasionally ☐ Never

10. Do your patients have a dedicated contact person in care?

☐ Always ☐ Often ☐ Sometimes ☐ Occasionally ☐ Never

11. How would you overall assess the quality of care for the patients at your workplace?

☐ Optimal care: Participation of the affected individuals and their families in setting goals, planning, and evaluating the care

☐ Adequate care: Consideration of personal habits.

☐ Safe care: routine care

☐ Unsafe care: frequent occurrence of avoidable complications

☐ Don't know

12. Have you ever cared for people with Parkinson during your entire caregiving experience?

☐ Yes, currently ☐ Yes, previously

☐ Never ☐ Don't know

Please add: ☐ approximately ___ people with Parkinson per week with on average ___ minutes nursing care time per day per person

☐ I cannot provide any further information about that

13. Is there a specialized care unit for people with Parkinson in your facility (priority care)?

☐ Yes, please explain: ____________

☐ No, but desirable ☐ No, no need ☐ Don't know

14. How do you assess the current nursing care situation for people with Parkinson in your facility?

Insufficient Optimal

☐0 ☐1 ☐2 ☐3 ☐4 ☐5 ☐6 ☐7 ☐8 ☐9 ☐10

☐ Don't know or I do not care for people with Parkinson's Disease

15. Based on your experience: In which areas/for which activities do people with Parkinson generally need the most nursing support?

- ☐ Transfer
- ☐ Personal hygiene
- ☐ Continence
- ☐ Dressing
- ☐ Food preparation or intake
- ☐ Going to the toilet
- ☐ Mediation intake
- ☐ Psychosocial consultation
- ☐ Buying /housekeeping
- ☐ Verbal and non-verbal communication
- ☐ Arrange appointments
- ☐ Reminding s.o. of sth.
- ☐ Handling of device-aided therapies
- ☐ Don't know
- ☐ Others

16. Do you think that the care of people with Parkinson is more complex and/or more time-consuming than that of other patients?

- ☐ Always
- ☐ Often
- ☐ Sometimes
- ☐ Occasionally
- ☐ Never
- ☐ Don’t know or I do not care for people with Parkinson’s Disease

17. Based on your experience: How precisely can / could timely administration of Parkinson's medications be maintained in your facility?

Not at all — Very precisely

☐0 ☐1 ☐2 ☐3 ☐4 ☐5 ☐6 ☐7 ☐8 ☐9 ☐10

☐ Don’t know or I do not care for people with Parkinson’s Disease

18. How in-depth is your knowledge regarding …

… symptoms of PD?

No knowledge — Very profound

☐0 ☐1 ☐2 ☐3 ☐4 ☐5 ☐6 ☐7 ☐8 ☐9 ☐10

… PD medication?

No knowledge — Very profound

☐0 ☐1 ☐2 ☐3 ☐4 ☐5 ☐6 ☐7 ☐8 ☐9 ☐10

… (handling of) deep brain stimulation?

No knowledge Very profound

☐0 ☐1 ☐2 ☐3 ☐4 ☐5 ☐6 ☐7 ☐8 ☐9 ☐10

… (handling of) PD medication pumps?

No knowledge Very profound

☐0 ☐1 ☐2 ☐3 ☐4 ☐5 ☐6 ☐7 ☐8 ☐9 ☐10

19. How important do you consider it that there is at least one specially trained Parkinson care professional in your facility?

Not important Very important

☐0 ☐1 ☐2 ☐3 ☐4 ☐5 ☐6 ☐7 ☐8 ☐9 ☐10

☐ Don’t know

20. Do you know about Parkinson-specific training options? Please tick at least one box for each question (multiple answers allowed).

	Never heard about	Heard about	Available in my institution	Participated myself
Parkinson Nurse (PD Nurse)	☐	☐	☐	☐
Parkinson assistant (PASS)	☐	☐	☐	☐
Parkinson Care specialist	☐	☐	☐	☐
Online School for Parkinson care	☐	☐	☐	☐

21. What suggestions do you have for improving the nursing care situation?

☐ More interexchange between patients and physicians

☐ More interexchange between patients and nursing staff

☐ More interexchange between nursing staff and physicians

☐ More interexchange with therapists

☐ More house calls from physicians to PD patients

☐ Use of telemedicine

☐ Constant nursing trainings on PD

☐ Constant trainings for therapists on PD

☐ Constant trainings for family doctors on PD

☐ More care time for PD patients

☐ More nursing staff in general

☐ Nursing staff specialized in PD

☐ Certificates for specialized trainings

☐ State recognition of nursing staff specialized in PD

☐ More competencies for nursing staff specialized in PD

☐ Better compensation of (specialized) nursing staff

☐ Interprofessional cooperation

☐ More priority care for PD patients

☐ Other suggestions

☐ Don't know or don't have any suggestions
